# Supplementary material for: Supporting adult unpaid carers via an online dancing intervention: A feasibility/acceptability study
Source: PLOS Glob Public Health. 2024 Jan 19;4(1):e0002798. doi: 10.1371/journal.pgph.0002798 (PMC10798499; doi:10.1371/journal.pgph.0002798)
Supplement: Attachment — Submitted filename: Response to Reviewers.docx [file pgph.0002798.s001.docx]

Dear Jianhong,

Many thanks for the considered feedback from your reviewers and for inviting us to submit a revised manuscript. We appreciate the time taken by the reviewers to provide such positive, comprehensive and constructive feedback. We have revised the manuscript, taking on board these comments, and feel that this has made a significant improvement to the article.

I have responded to each comment in turn below and provided line numbers where the revisions can be found [**NB These line numbers relate to those in the Tracked Changes version of the manuscript**, which are different to those in the non-tracked manuscript due to a known bug in Word].

Best wishes,

Jo.

Reviewer #1: The paper is very well written. It is clear and concise and well referenced. Thank you.

The introduction is excellent. The authors have set out the context of the physical and mental health problems experienced by unpaid carers and the benefits of, and barriers to, increasing their physical activity. The purpose of the research is clear. This is an interesting and potentially important intervention for unpaid carers. Thank you.

The methods section is again well written and clear but it is missing a section on research design, looking at the pros and cons of undertaking qualitative semi-structured interviews with a sample of unpaid carers. I agree with the research design but it would have been good to see a justification for it. This information has been added (lines 121-131).

I would have liked to read something about the intervention itself. What sort of dance? Was there a choice of style? Could the carers take part in the live class and also have access to the pre-recorded video if they wanted to watch it again or do the session again? This information has now been added (lines 180-218).

The section on data analysis is overly brief. I would like to read more about how you analysed the data - was it transcribed and coded manually? Were transcripts (or a sample of them) coded by more than one researcher? How did you analyse the coded data? Also the Braun and Clarke reference is quite old now. There are new materials by Braun and Clarke which reflect their further thinking around thematic analysis. This information has now been added (lines 228-242).

Again, the section on ethical considerations is overly brief - was the care recipient present during the pre and post interviews or was the carer advised/able to be interviewed in private? Were there any incentives for participants to take part (e.g. a shopping voucher as a thank you)? Did potential participants have the opportunity to ask questions ahead of the interviews? This information has been added (lines 257-260 and 268-272).

Also, did some carers take part in pre-intervention interviews but not post-intervention interviews? One participant could not complete the post-intervention interview – this has now been added in the Procedure (lines 220-221).

The results are very interesting, though very descriptive. I don't understand how participants were familiar with the dance instructor from the pre-intervention interview - was the interviewer also the dance instructor? This has now been explained in the method (lines 171-178).

The discussion is brief but good and contextualises the findings within the wider literature. However, I would like to see a limitations sub-section in the discussion - perhaps considering carers who are not computer/internet literate and/or those who do not have the physical space to dance in their own homes? A limitations section has been added (lines 895-911).

Perhaps before you talk about the expansion of the programme and making online dancing classes a provision regularly offered to carers, it would make sense to first test the intervention to see if/to what extent it actually does improve mental and/or physical wellbeing and also how the intervention works for carers of people with physical care needs or learning disabilities etc. This has been added into the limitations section and the sentence in the conclusion amended (lines 904-911 and 914-915).

There is a good range of relevant and recent references and the referencing style is excellent. Thank you.

Line 31, insert the word 'between' before 21%. These are separate figures for physical / mental health – I have made this clearer (lines 31-32).

Line 445, suggest remove the words 'in this manuscript'. Done (line 853).

Line 501, should 'or' be 'of'? Corrected (line 944).

Line 513, 'o flongitudinal' should be 'of longitudinal'. Corrected (line 956).

Reviewer #2: I really enjoyed reading this interesting paper about an online dancing class as an intervention to increase carers’ physical activity- these sorts of interventions are much needed and this one showed some promise. If the issues shown below are resolved, I think it would be an interesting and useful paper. I have listed some major overall points and then some issues line by line. Many thanks.

1. You need to be clearer that the research us in the UK and describe the UK context. Be clear when you cite UK-based research compared to international research. How could this intervention be used around the world? This is a Global audience. Which populations might it be useful for outside of the UK? UK-based research has now been made explicit (lines 16-17, 31, 61, 67, 69, 79, 83, 95). The use of a UK sample has been added into the limitations section (lines 897, 904-906).

2. You need to be clear when talking about Covid-19 pandemic and the policies that resulted from the pandemic – such as, self-isolation and limited contact. Each country had a unique story and you must be clear about the UK context. This information has now been added (lines 83-90).

3. There is a potential ethical issue arising from the dance teacher also being an author – you just need to be transparent about their involvement and how you mediated any potential bias. The subjective position of all researchers has been added (lines 243-251).

4. I wasn’t sure how the intervention was developed – this seems an important aspect considering it is a feasibility study This information has now been added (lines 184-218).

5. Need more information about recruitment/who got the email/ any pre-existing links to Brighton carers centre etc This information has been added (lines 159-165).

6. The use of thematic analysis is partly incorrect and is confusing- please see my comments below. Tightening up this part of the paper would improve it a lot. At the moment all the ‘themes’ and ‘subthemes’ are confusing- especially in the abstract. The data is so interesting – but it is masked by the confusing write up. Some of the themes are mislabelled themes- when really they are questions. Think about what themes are – you definitely have some themes but also just have some responses to questions in there- which is fine! We agree that the original themes are essentially questions – we have reconsidered the themes in light of this and new themes have developed – these have been amended in both the abstract (lines 18-21) and results sections (entire section).

7. Be careful when making assumptions about mental health carers and their caring loads. Wording has been amended (lines 528-533).

8. Needs to connect to the wider literature on a) different types of carers b) online exercise or group interventions and their barriers and facilitators c) who joins these interventions and who might be excluded. This is missing in the opening and the discussion. There is a whole literature out there on online interventions – and how they have been used since the pandemic. And on different types of carers and who engages and who doesn’t – and who does or doesn’t take part in physical activity etc. Need a lot more on this. This has been briefly referred to in the Introduction (lines 97-101) and discussed in depth in relation to the findings of the study in the Discussion (lines 641-851).

9. Reflection on how to broaden engagement and participation to address the clear issues in the heterogeneity of your sample, or some discussion about this. This has been added within the limitations section (lines 896-911).

10. Finally, the whole paper could do with a good edit – there are some bits that don’t make sense or could be a bit better explained. I have tried to point these out as I came across them. All points addressed below

Line by line:
Abstract- needs to state it is the UK. Done (lines 16-17).
LINE 14 – not sure about the word ‘suffer’ – experience may be better Corrected (line 30)
Repetitive abstract – ‘revealed two key themes’ Removed (line 21)
Really not keen on the word suffer-try to choose different words. Corrected (lines 33, 35)
Line 44- not clear this research is in the UK or what methods used Added (line 67)
Line 45- same. Added (line 69)
Line 51-52 leisure time physical activity’ -maybe say this better? Explanation added (lines 74-75)
Line 54- ‘Barriers to physical activity include increasing aging’ – this is a bit ageist –What do you mean by this exactly? The link between ageing and health/mobility? Clarification added (lines 79-80)
Line 56-57 – Again make this clear it is the UK context – be specific it is research by Carers UK etc Done (line 83)
Line 56 Could be a new paragraph start, following with line 59 & 60. Agreed – changed (line 83)
Line 59. Think you need to explain the pandemic a little bit more- make it clear the UK context as this is a global health journal. Added context
Line 61 ‘Increased demand on coping’ isn’t very well written Amended (line 91)
62- ‘It wasn’t impossible to take part in group exercise classe’s- it was actually very common in the pandemic- online. It was actually very common. Be clearer. Made it clear that this refers to face to face classes (line 94)
65- during future lockdowns? Amended (line 97)
71- “in non-quarantine periods” is a strange term- you could say, in normal times… etc.. Or link into arguments about how the digitalisation of interventions during Covid-19 pandemic has resulted in a changed landscape for UK service delivery. There are some papers circulating on this topic now- long lasting impacts of Covid-19 pandemic. Amended (line 107)
76 – do you mean topic guide – not familiar with the word schedule? Amended (line 132)
90-91 Emailed to who?- How did you gain access to Brighton Carer Service? What is the Brighton Carer Service- what does it do? Explain for a non-uk audience. What types of carers are registered. Information added (lines 159-162)
97- Who curated this dance activity? How? Was it evidence informed? How long did it take? Was it piloted? More about the intervention please. This information has now been added (lines 184-218).
108-109 – More information on data analysis is needed here. Who analysed the data? How? Why Braun and Clarke’s model? This information has now been added (lines 228-242).
117- The dance teacher was a co-author? I feel like more needs to be made of this. I think this raises an ethical question in itself. You need to be more transparent about their exact role in the study, how you mediated for any potential bias etc. The potential for bias is now covered in the data analysis section (lines 243-241). The authors prior connection to the Brighton Carers Centre. This is now stated in the Procedure section (lines 161-162). The dance teacher- did she know any of the participants before the study through the dance community? Etc. Did the authors know any of the participants in the dance classes before the study? It is fine if you do- but that needs to be explained and taken into consideration. This has been added under Participants (lines 155-157).
129- The way you’ve talked about this as themes seems incorrect for me – For example, Theme 2 in Table 1- I think there are three themes in response to the question on expectations. But expectations isn’t a theme itself- it is an interview question.
Similar applies across all themes.
Theme 1 could be – in response to why they wanted to take part themes were identified which could be 1 and 2. But that isn’t a theme? It is a subset of questions.
Again- apply to 3.2 – the use of themes isn’t right- a theme isn’t an interview question/topic. It is how you organise the information in the responses to a question/topic. We agree that the original themes are essentially questions – we have reconsidered the themes in light of this and new themes have developed (entire Results section has been amended).
200- mental well-being side of health, is a clumsy expression. Amended (line 373)
220. Why did someone drop out? You say 5 here. This has now been explained in the Procedure (lines 220-221).
272- Individual style particularly likeable- could you say, individual style was liked by these participants? Amended (line 476)
355- be careful with how you refer to mental health carers and the assumptions you make here about the challenges of being a mental health carer and their availability. Mental health caring can be just as challenging and lead to the same lack of time. This sentence has been removed and the following sentence made clearer regarding the impact of caring on fatigue and time (lines 528-533).

Other comments.
Think about- who didn’t come forward? Who didn’t volunteer? This is considered in the Discussion (lines 641-851) and under the limitations section (lines 896-899, 904-909.

Need more info about how this research could be generalised/transferred to other contexts etc This has been referred to in the limitations section (lines 904-906).

I want to know how many of the participants did each class and what method they used? Online/offline This has been added into the Procedure (lines 181-184)
